# Psychological safety in interdisciplinary teams: how leadership behaviors empower teams

**DOI:** 10.3389/fpsyg.2026.1768461

**Published:** 2026-02-25

**Authors:** Stephanie M. Resendiz, Maria Hernandez, Molly Murphy, Shannon Casey, Michelle A. Chui, Elizabeth S. Burnside, Whitney A. Sweeney

**Affiliations:** 1 Division of Clinical Practice, Innovation, and Research, School of Pharmacy, University of Wisconsin-Madison, Madison, WI, United States; 2 Institute for Clinical and Translational Research, School of Medicine and Public Health, University of Wisconsin-Madison, Madison, WI, United States; 3 School of Nursing, University of Wisconsin-Madison, Madison, WI, United States

**Keywords:** leadership, psychological safety, team culture, training and education, translational teams

## Abstract

**Introduction:**

Interdisciplinary collaboration drives innovation by uniting diverse knowledge and perspectives. However, bias and ineffective team management practices can hinder a team’s ability to leverage these advantages by causing interpersonal conflict, eroding trust, and compromising communication. These challenges reinforce the need for leadership skills that extend beyond team management practices that focus on coordination and task execution, emphasizing the relational work required to cultivate healthy team dynamics. By equipping leaders with tools to foster stronger team cultures, teams are better positioned to integrate unique perspectives, unlocking the full potential of their collective expertise.

**Methods:**

Taking a phenomenological approach, eight senior leaders with extensive experience in leadership and mentoring were interviewed to learn more about their experiences in promoting psychological safety in teams. Transcripts were coded using thematic analysis during multiple coding iterations.

**Results:**

The behaviors described by interviewed leaders fell into five thematic areas: self-awareness and growth, mentorship and development, amplification and empowerment, communication and transparency, and accountability and integrity.

**Discussion:**

This study underscores the transformative power of leadership in cultivating strong team cultures while also complementing and strengthening existing leadership-training efforts. Leadership development programs could be strengthened by attending more explicitly to psychological safety, and our findings provide actionable insights that can be integrated into current training frameworks to deepen leaders’ capacity to foster supportive and high-functioning teams. Ultimately, this shift not only benefits individual teams but has the potential to strengthen the fabric of the broader research culture.

## Introduction

In today’s increasingly complex and interconnected world, research is more frequently conducted in teams, particularly those that span multiple disciplines, institutions, and geographies (Jones et al., 2008). Studies have shown that team-authored papers are not only more common but also more likely to be cited, suggesting they have a higher impact (Wuchty et al., 2007). Interdisciplinary collaboration fosters innovation by integrating specialized knowledge and perspectives from different fields, enabling teams to tackle multifaceted problems more effectively than individuals working in isolation (Mello and Rentsch, 2015; Wang et al., 2016). However, bias and ineffective team management practices can hinder a team’s ability to leverage these advantages by causing interpersonal conflict, eroding trust, and compromising communication (Carter and Phillips, 2017). These challenges reinforce the need for leadership skills that extend beyond team management practices that focus on coordination and task execution, emphasizing the relational work required to cultivate healthy team dynamics (Carmeli et al., 2010; Wang et al., 2016). By equipping leaders with tools to foster stronger team cultures, teams are better positioned to integrate unique perspectives, unlocking the full potential of their collective expertise.

This capacity to integrate diverse expertise depends heavily on psychological safety, which creates an interpersonal environment that makes such collaboration possible. Psychological safety, “the shared belief that the team is ‘safe’ for interpersonal risk-taking,” (Edmondson and Bransby, 2023) permits members to ask questions, be vulnerable, make mistakes, and challenge the status quo without fear of negative consequences (Clark, 2020; Edmondson, 2018; Edmondson and Bransby, 2023). Psychological safety is a team-level phenomenon that emerges as team members first build trust in the leader and then extend that trust to one another (Brasier et al., 2023b).

Over the past decade, research in psychological safety has expanded to reveal four interconnected domains that shape how teams function (Edmondson and Bransby, 2023). First, it enables people to get things done by fostering the open communication needed for effective coordination and performance in complex environments. Second, it supports learning behaviors, encouraging team members to discuss mistakes, ask questions, and experiment -- activities essential for continuous improvement. Third, psychological safety plays a central role in improving the work experience, creating an environment where individuals feel respected, included, and able to contribute authentically. Finally, leadership emerges as a critical driver, as leaders set the tone that allows psychological safety to develop through their behaviors, expectations, and responses. Together, these themes illustrate how psychological safety functions not just as a cultural ideal, but as a practical foundation for high-performing, adaptive teams (Edmondson and Bransby, 2023).

This foundation sets the stage for a more expansive understanding of leadership within teams. It is worth noting that leadership need not be confined to formal titles or specific positions. While the positional leader sets the tone and models the values that guide the group, teams thrive when individuals feel as though they can take initiative and contribute leadership from wherever they are within the team. When team members feel empowered to influence processes, raise concerns, and support one another’s success, leadership becomes a shared, distributed responsibility rather than a role held by a single individual (Brasier et al., 2023a). Thus, every team member has the capacity to shape team culture and enhance overall performance, making leadership a collective endeavor.

Across recent studies examining team functioning, a clearer picture emerges of how leaders and team members can actively cultivate psychological safety. Central to this process is the idea of trust, first between leaders and their followers, and then within the broader team. Trust develops when leaders demonstrate individualized concern and respect, recognizing each person’s work style, strengths, and needs (Brasier et al., 2023a). By modeling trusting norms, leaders signal that it is safe for others to do the same, helping shape an emotionally supportive team climate.

Building on this foundation, other researchers have highlighted the importance of intentional team formation and facilitation practices that reinforce supportive norms (Jones et al., 2024). Effective leaders and facilitators create conditions where members feel comfortable taking risks, asking for help early, and openly sharing expertise, which helps teams leverage the unique skill sets of their members. These leaders and facilitators also normalize discussion of problems and mistakes, demonstrating that these moments are opportunities for learning rather than threats. Preventing social undermining, such as dismissiveness or subtle exclusion, emerges as another essential practice, as it protects collaborative relationships and reduces interpersonal threat (Jones et al., 2024).

TeamMAPPS (Team Methods to Advance Processes and Performance in Science) is an evidence-based team science training program designed to strengthen the behavioral competencies that enable effective collaboration in scientific teams (Bisbey et al., 2021). Central among these competencies is psychological safety, which TeamMAPPS positions as the essential interpersonal foundation for high-quality teamwork. Rather than treating psychological safety as a passive cultural attribute, the program frames it as a set of deliberate, learnable behaviors that cultivate a climate in which team members feel able to ask questions, raise concerns, share unique insights, and acknowledge uncertainty without fear of embarrassment or negative consequences. Through its emphasis on practices such as acknowledging contributions, surfacing issues constructively, and navigating conflict respectfully, TeamMAPPS underscores that psychological safety is the mechanism that unlocks open communication, fosters intellectual risk-taking, and supports the integration of diverse expertise within scientific teams (Bisbey et al., 2021).

Taken collectively, current research (Bisbey et al., 2021; Edmondson and Bransby, 2023; Jones et al., 2024) suggests that psychological safety is not passive, it grows through trust-building leadership, skilled facilitation, and deliberate training experiences like TeamMAPPS that cultivate the relational competencies teams need to collaborate effectively. When these elements work in concert, teams become more open, resilient, and capable of leveraging their full collective expertise.

This project aims to extend the existing evidence base while strengthening current initiatives (Bisbey et al., 2021; Brasier et al., 2023a; Sweeney et al., 2023) that train researchers in the skills needed to create and maintain psychologically safe team environments. Taking a phenomenological approach (Patton, 2015), eight senior leaders with extensive experience in leadership and mentoring were interviewed to learn more about their experiences in creating supportive team cultures. Rather than learn from leaders with backgrounds that reflect dominant cultures exclusively, leaders whose perspectives were shaped by navigating exclusion within the scientific community were selected. The goal was to learn more about the successes and challenges these leaders experienced when creating psychological safety in their teams and identify the actions they took during the process. In addition to identifying specific actions as individual leaders, we were also interested in how they inspired other team members to promote psychological safety. This study seeks to generate actionable insights to inform a workshop curriculum that builds upon, and complements established team science training resources. This added learning opportunity will support leaders in cultivating psychological safety, strengthening team cultures, and encouraging others to lead in similar ways.

## Materials and methods

The current study involved three steps: (1) creating and consulting an Advisory Board, (2) qualitative interviews and thematic data analysis, and (3) consensus building and translating the findings into practical recommendations. Four doctoral-level researchers and two students (one doctoral level) conducted the study. One researcher left the project after the interviews were completed, due to circumstances unrelated to the project. An Advisory Board made up of an additional three experts in higher education programming was convened. Advisory boards are increasingly used in qualitative research studies to help improve methodological rigor and provide additional support for interdisciplinary collaboration (Faissner et al., 2025). The Advisory Board met three times prior to conducting interviews, and again at the end of the data analysis phase.

### Sampling and recruitment

Purposive sampling was used to create a sampling frame of senior faculty members who had expertise in leadership and mentoring, were affiliated with clinical and translational research, and were employed by the same large Midwestern higher-education institution. All participants were affiliated with the local Clinical and Translational Science Award (CTSA) and as a result were part of a broader research community. All participants were principal investigators (PIs) of clinical or translational/biomedical research teams. Those identified as eligible to participate were sent an email invitation in early summer 2024 to participate in semi-structured interviews. A total of eight (*N* = 8) senior faculty members were invited to participate in the study, and all consented to participate. The senior faculty members held the title of Associate Professor (*n* = 3) or Professor (*n* = 5). Participants were affiliated with the schools of pharmacy (*n* = 5) and medicine and public health (*n* = 3), with one participant having a joint affiliate role with Veterans Affairs (VA). In addition, the majority of participants also held higher level administrative titles (e.g., chair, director, or assistant director). This study was reviewed by the IRB and deemed exempt (ID# 2024–0871).

### Data collection and analysis

Qualitative interview questions were developed by the research team with the intent of serving as a needs assessment to inform workshop curriculum designed to help train leaders to promote psychological safety in their teams. The initial interview guide was presented to the Advisory Board for feedback, which led to the development of the final iteration of interview questions. Questions aimed to explore ways to promote psychologically safe environments from the perspectives of study participants. More specifically, the interview guide was structured in the following way:

- **Welcome and introduction**—This section started by describing the study and asking participants to share a bit about themselves, specifically in the context of serving as a PI/leader in clinical and translational/biomedical research.
- **Team environment**—This section contained questions that were designed to learn more about how participants created strong and psychologically safe team environments. Follow up questions addressed how participants encouraged others to do the same.
- **Challenges**—This section asked participants to describe the ways in which they had struggled to create strong and psychologically safe environments.

A staff member trained in qualitative methods conducted semi-structured interviews lasting approximately 45 min with all participants via Zoom (Zoom Video Communications Inc., 2025). Interviews were audio and video recorded using the Zoom platform. Although the interviews were conducted by a staff member rather than a graduate student or faculty researcher, we intentionally incorporated mechanisms to address potential power dynamics. The interview guide was reviewed and refined by the advisory board which included senior researchers who were familiar with the institutional context and the hierarchical dynamics typically present in faculty-staff interactions. Their involvement helped ensure that questions were appropriate, respectful, and unlikely to place participants in a vulnerable position. At the same time, the interviewer’s status as a staff member (someone positioned outside the traditional faculty hierarchy and typically viewed as providing support rather than evaluation) may have mitigated some power imbalances that can arise when more junior researchers interview senior faculty. In several respects, this positioning allowed the interviewer to step outside normative academic rank structures, potentially fostering a more open, less guarded exchanged during the interviews.

Interview transcripts were initially generated by Adobe Premiere Pro (Adobe Inc., 2025), then verified by the research team who corrected and confirmed the accuracy of the transcripts. Our process is aligned with the six-phase process for thematic analysis outlined by Braun and Clarke (2006): familiarizing yourself with your data, generating initial codes, searching for themes, reviewing themes, defining, and naming themes, and producing the report. Two research assistants (RAs) (MH & SR) coded the interviews utilizing thematic analysis (Braun and Clarke, 2022). We used a combination of deductive and inductive coding during multiple coding iterations. The first phase of analysis followed a deductive approach in which the interview-guide questions served as the initial code set. That is, each question was operationalized as a code and applied systematically to identify portions of the transcripts addressing the question topics.

A second pass utilized another iteration of deductive coding, which helped categorize the general themes the RA’s extracted from the initial pass. Our second analytic pass involved clustering the data into three overarching categories. One theme captured the challenges interviewees identified in creating strong team cultures. The other two were derived from Roberson and Perry’s (2022) conceptualization of leadership and focused on leader mindset and behaviors adding team mindset, respectively.

In the final round of analysis, we conducted an inductive coding pass focused on isolating the actionable behaviors participants described across the second-pass themes. We treated thematic saturation as the point at which no new leadership actions, strategies, or practice-oriented elements were emerging from additional interviews. Once the data yielded only reiterations or elaborations of existing behaviors, we considered the set sufficiently saturated to inform curriculum development (Patton, 2015).

To ensure a transparent and trustworthy analytic process, we used team-based coding and iterative consensus building across all phases of analysis. During the first two rounds of coding, the RAs independently coded the transcripts and then shared their coded segments with the broader research team (MH, MM, SC, SR, & WS). These coded datasets were reviewed collectively in weekly meetings over 2 months where team members engaged in critical interpretation, questioned assumptions, and resolved discrepancies through discussion until consensus was reached. In the final phase, specific leadership behaviors and actions within each theme were inductively identified. The refined set of codes were shared with the entire research team. The group again confirmed the coding decisions, ensuring that the finalized code set reflected shared agreement and analytic rigor.

Our analytic approach was informed by ongoing reflexive attention to the identities, backgrounds, and positionalities represented on the research team. The team included two student research assistants, both of whom brought perspectives informed by unique cultural experiences. The rest of the broader team included three professional staff researchers with PhDs and two faculty members in senior leadership roles, whose institutional positions and career trajectories shaped how we understood issues of authority, mentorship, and power raised by participants. Throughout analysis, we engaged in reflexive dialogue to consider how our varied social identities, institutional roles, and lived experiences informed our sense-making and interpretations.

## Results

Below we present the results of our thematic coding. This section describes the behaviors that experienced leaders used to foster strong team cultures and promote psychological safety within their teams. The behaviors described fell into five thematic areas described below (see Table 1).

**Table 1:** Leader behaviors that promote psychological safety in interdisciplinary research teams.

Thematic areas	Leader behaviors	**Self-awareness and growth** Behaviors that promote self-reflection and continuous self-improvement	Pursue formal leadership training (e.g., workshops) Engage in peer-to-peer support networks and communities of practice Hire a professional coach to develop specific leadership behaviors Adopt a growth mindset to learn from personal challenges Practice self-reflection to develop awareness of reactions and uncover biases
**Mentorship and development** Behaviors that nurture growth and career advancement in team members	Create opportunities for team members to get to know each other (e.g., ice breakers, lunches, and retreats) Initiate one-on-one conversations to better understand individual needs and strengths Encourage and support opportunities for professional development Share individual lessons learned with team members Seek guidance from experienced peers and support junior team members
**Amplification and empowerment** Behaviors that promote shared leadership and integration of unique perspectives	Model vulnerability by sharing appropriate personal experiences and insights with team members Develop shared team norms that encourage the expression of varied viewpoints Actively seek expertise from all team members including junior team members and community partners Create supportive team partnerships by grouping individuals with complementary skills Celebrate team successes and reinforce a shared commitment to helping everyone shine
**Communication and transparency** Behaviors that encourage openness, clarity, and shared understanding	Ensure everyone has common understanding of jargon and constructs Encourage feedback from team members Provide opportunities to voice concerns and ask clarifying questions Ask team members about what they need to feel supported Curate sets of research articles to ensure shared understanding of relevant topics, bridge the gaps between disciplines, and lessen the hierarchy created by titles and ranks
**Accountability and integrity** Behaviors that support fairness, consistency, and follow-through	Create team environment in which members can share equally and listen respectfully Find common ground during conflict by revisiting the shared vision and values Watch for harmful behaviors, address concerns directly and encourage others to speak up Disagree without being disagreeable by providing alternative viewpoints in a spirit of mutual respect Support team members during challenging moments by regularly checking in on their comfort and confidence in tasks and offering help when needed

### Self-awareness and growth

The first thematic area highlights leaders’ commitment to self-reflection and continuous improvement. Many pursued ongoing learning through literature, workshops (e.g., leadership), and learning communities to build skills such as self-awareness, active listening, and perspective taking. Peer-to-peer learning also emerged as valuable; whether through formal programs (e.g., K scholar programs) or informal networks, leaders valued having a safe space to exchange ideas, share challenges, and explore solutions.

*“I can bounce ideas off them (…), I can be vulnerable and stupid and say, I made a mistake, or I should have done this different. And they’re incredibly supportive.”*

Several leaders highlighted professional coaching as an important support for developing leadership skills, particularly when taking on more advanced roles. Coaching’s emphasis on reflexivity stood out; one leader noted that she now conducts weekly self-check-ins to review her goals and incorporate team feedback.

*“I have meetings with myself about my leadership, and then I go back to my own goals, restructure what I need to, analyze how I’ve done, and then I get feedback also from my team. So, I incorporate feedback from my team …”*

Leaders underscored self-reflection as key to recognizing bias and micro-aggressions, illustrated by one interviewee’s account of harm they experienced during a presentation.

*“I'm giving this presentation about all the things that the research center has been doing. And at one point the funder (…) interrupted me and said something like, “Well, young lady, blah, blah, blah, blah, blah.” And, you know, I recognized it immediately as a microaggression right and I left the room and my division chair who left the room with me … soon as we closed the door, I mentioned this to him, and he had completely missed it.”*

The participant described feeling isolated when others overlooked the incident until she named it. Another participant noted that such experiences often require time and reflection to fully “unpack.”

*“But you know, even growing up, you know, I would get these things and it causes for me at least, self-reflection for very long periods of time to try to unpack why these things happen and try to like rebuild yourself back, back up to where you were before.”*

Through self-reflection and continuous self-improvement, interviewed participants were better able to model what they had learned from their personal experience and the training they pursued to better promote psychological safety on their teams.

### Mentorship and development

The second theme focuses on behaviors that promote team growth and advancement. Leaders highlighted the value of connection, often built through one-on-one meetings that surface team members’ preferences and skills. One leader, for instance, meets individually with new hires during onboarding.

*“… I really work to develop a one-on-one relationship with each team member. So as part of their onboarding, I would meet with each team member often, two, maybe three times to develop a ground rules document. In that ground rules document, it was more than just like, “Hey, here’s the expectations.” It’s more there were questions like, … What do you enjoy about working in a team? What are the tasks that you’ve done on other teams that you like? What are tasks among the teams that you dislike? You know, so. So, it was really more about learning about the person (…) which I think was really helpful because before we had larger team meetings, they, the individuals sort of knew me and they had an idea of what we would be working on and how they could contribute.”*

One-on-one meetings were described as tools for setting expectations and discussing difficult topics. It provided an opportunity to address conflict as opposed to letting it “simmer” or “stew.”

*“…don’t mull it over in your head and upset yourself and get stressed out. Let’s just talk it through.”*

One participant emphasized that icebreakers (e.g., asking about weekend plans or current books) are effective ways to learn about team members. Another added that starting meetings with quick check-ins can build “human capital.” Other leaders shared that organizing celebrations, informal lunches, and retreats also fosters connection and team cohesion.

*“So, but it was fun to watch, you know, favorite Halloween candy. Somebody could be like, they like the candy that I like. Okay, there's a little human connection there. You know, it's, it's nothing earth shattering, but I feel like it helps reorient them to the fact that we all have the same mission. We're all human. We're all doing the best we can.”*

Relationship building is rooted in authentic, two-way exchanges and some leaders described the importance of sharing personal information with their team members during team activities as an equal participant. One participant even described how she shares when she is struggling with her team.

*“And, you know, if I’m struggling with something, I’m actually frequently deliberately put that out there and say, well, okay, so here’s the situation. This is what I really want to do, but I don’t know if it’s feasible. What do you guys think? How can I make this work? Can I make this work? Is this something that’s not worth the, you know, not worth the effort? What do you think? And so, I, I deliberately, you know, try to make relationships and information exchange two ways.”*

Leaders described how modeling humility and seeking input fosters reciprocal trust and richer perspectives, helping them more effectively support professional growth. They also underscored the value of investing in junior colleagues. One leader spoke of senior staff “paying it forward,” while another noted that the mentorship she once received now shapes her approach to leading her team.

*“(…) but one thing I would never (…) forget is my advisor played a critical role in the mentoring that I received and the support that I received. And when I first started my career, I guided my mentoring based on what I learned from her…”*

Participants stressed that one-on-one interactions build trust and clarify team members’ strengths, preferences, and challenges. Informal practices like icebreakers, check-ins, and celebrations further supported cohesion. By modeling humility and drawing on their own mentorship experiences, leaders fostered open dialogue and “paid it forward” to support the next generation.

### Amplification and empowerment

The third thematic area highlights behaviors that foster shared leadership and elevate diverse perspectives. Leaders described how some individuals, particularly those who were junior in rank, hesitated to fully engage in group settings due to fears of not being accepted, appearing unprepared, or challenging those with positional authority. Several interviewees noted that even tenured faculty sometimes felt unsafe contributing, underscoring how identity and power dynamics shape team interactions. Recognizing this, leaders became more attentive to creating environments where all team members could “feel seen.”

*“I’m a full professor. I’m tenured. (…) and yet still I don’t feel safe, which means that if I don’t feel safe, I’m making an assumption that virtually no one else feel safe. So, I do sort of take that in thinking about my own positionality and [PAUSE] how I think about trying to support others who I perceive also don’t feel safe, probably less safe than me, even.”*

To reduce barriers to participation, many leaders established private avenues for questions and concerns, such as text messages or email, which helped alleviate fears about speaking up publicly.

*“And for some of my graduate students, they’re afraid to ask questions (…) so they’ll actually text me in real time the question because they are afraid to ask it aloud.”*

Other leaders modeled vulnerability or realistic expectations to counter hierarchical norms in academia, where colleagues may not be accustomed to “taking care” of one another. These actions helped signal authenticity, approachability, and psychological safety.

*“We’re all sort of like independent contractors of maybe rowing in the same direction (…) that experience has really colored my thinking about how I feel like I need to take care of other people how I feel like no one should be in a situation like that, like I was where I felt like I had no power and I felt like I was I was caught where I didn’t have another option to get out of the situation and still achieve what I wanted to achieve.”*

Creating intentional spaces for idea-sharing emerged as a central strategy. Leaders described inviting quieter team members into discussions to ensure their insights were not overlooked. Some paused thoughtfully to consider questions, even when misaligned with the topic, and offered clear explanations for their decisions. This kind of listening, without judgment or distraction, was described as essential to building confidence, mutual respect, and honest dialogue. For one leader conducting community-based participatory research, recognizing, and empowering the expertise of community partners exemplified this commitment to shared leadership.

Honoring team members’ expertise began with making space for their voices, but leaders also emphasized the importance of sharing leadership itself. Some created opportunities for team members to take on leadership roles or guide decision-making processes. Others used meeting structures to reinforce collaboration, for example, circulating draft agendas for team input or allowing team members to generate the agenda entirely, ensuring that everyone had a voice.

*“Every single person contributes to that agenda because we want people to feel like I have a voice. I also have something to say. (…) Well, that's changed the dynamic of our team meeting so much. When I wasn't the leader of the meetings, I wasn't the one calling people to speak… And it was very intentional. Where even in our team meetings I would maybe not even speak. I would start with, “Okay, (…) what are we talking about today? And she leads and then she takes charge of everything.”*

Leaders also described how their own experiences informed them of their efforts to support others. Many embraced their positionality to promote psychological safety, including one leader who ended meetings by asking, “How do you feel about that?” Such reflective questions surfaced concerns early and signaled a commitment to shared ownership of decisions.

*"So whenever I'm meeting, (…) I always end with “How do you feel about that?” “How do you feel about this?” I use that question a lot and it's… it's pretty effective at getting at their comfort level. You know, it's not about getting the task done. It's about their confidence or their like how equipped they feel to get the job done."*

A consistent theme across interviews was the importance of supportive teams grounded in collective well-being and shared achievement. Leaders described helping team members learn from failure, develop new areas of expertise, and rely on one another for mutual assistance. Several described fostering a “safe failure” culture where experimentation and professional growth were encouraged. In the strongest examples, this foundation of trust led to environments where colleagues championed each other’s successes. As one leader explicitly told her team, she wanted “everyone to win, everyone to shine.” Another shared her belief that every team member had the potential to “change others,” which guided her efforts to cultivate belonging.

### Communication and transparency

The fourth thematic area centers on behaviors that encourage openness, clarity, and shared understanding. Leaders emphasized creating environments where team members could fully engage and even share leadership responsibilities. A key aspect of this was clarity in communication, as one participant described, “making the implicit, explicit.” Leaders worked to not only articulate their own expectations but also to create opportunities for team members to express their needs through frequent, frank conversations.

*“…I think almost every week we have something that kind of comes up of where we have some frank discussions with people about how they’re doing, how we can help them, how they can evolve to new roles and to take risks and doing kind of doing some self-searching, …”*

Regular team meetings served as important spaces to keep everyone informed, minimize misunderstandings, and provide opportunities for questions and discussion. Some leaders highlighted the value of “open communication” and intentionally sought out their team members’ perspectives during meetings.

*“… You can sit there quietly until I call on you, and then I want to know what you’re thinking. And I know you’re quiet, but I still want to know what you’re thinking, which makes people uncomfortable. But, you know, sharing what you’re thinking is an important part of being on the team…”*

Leaders also recognized the importance of establishing a common understanding of terminology and constructs. Some terms, such as *psychological safety*, were seen as intimidating or unclear. One participant even described the phrase as “mumbo jumbo” with associated stigma, noting that team members should not feel they must “walk on eggshells.” To address this, leaders emphasized unpacking and defining key concepts together: “You want to be frank and up front with clear communication.” By demystifying terms and making expectations explicit, leaders felt they fostered more psychologically safe team cultures.

*“So, I think, you know, I don’t even know that I use it with my team, but I think a lot of folks are like, it’s not my job to be your safety net, right? It’s not my job to make sure you feel safe, like it’s not dangerous here. What’s the danger here? Everybody’s safe. (…) And I should be allowed to disagree with you, Right? That notion about feeling like psychological safety means you have to agree with everyone and treat them with kid gloves. Right. And I don’t want to have to do that.”*

Feedback practices were another critical component of transparency and engagement. Leaders encouraged input from team members, even when busy schedules or hierarchy created barriers. Strategies ranged from end-of-semester reflections in student-heavy teams to collaborative review of lab manuals. Leaders found this feedback invaluable for refining their practices and deepening relationships, connections that in turn supported more honest and meaningful dialogue. Some leaders used quick tools like a “five-minute summary” to gather frequent updates, needs, and successes.

*“And so now all of my lab uses the five-minute summary (…) what I've been working on. what I need from you, and what's my win for the week. And it cannot take more than 5 minutes. It's (…) free associated, you don't have to write full sentences but the -- They just email it on Fridays.”*

Finally, leaders emphasized the importance of constructive dialogue in integrating diverse perspectives. Teams benefited from environments where members could share equally, listen respectfully, and “disagree without being disagreeable,” which one leader described as crucial for scientific progress. Returning to the team’s shared vision helped anchor discussions.

*“And it seems like sort of a tangible thing for me about creating psychological safety (…) is that there has to be some shared set of readings, I believe, because otherwise the degrees of training, not degrees of title, but the degrees of training will show up right on there. The humanities would clash with the social sciences and behavioral sciences and the basic scientists. So, we have to have a common script to be reading from and then we can poke on it.”*

This leader noted that ensuring members were “informed” by curating research articles helped everyone “see the same part of the elephant” and reduced disciplinary or hierarchical divides. These practices helped create a common language and a space where team members felt supported, even amid disagreement, as they worked toward collective goals.

### Accountability and integrity

The fifth thematic area highlights behaviors that support fairness, consistency, and follow-through. Interviewees recognized that their decisions had the potential to significantly impact the team, were aware of the consequences of their behaviors, and took ownership. Further, all leaders emphasized understanding that psychologically safe teams are those in which everyone is held accountable by aligning with shared values.

*“…we did an activity on the board about values-based decision making that I thought was really, really helpful (…) I still go back to when I’m dealing with challenging things, you know, what are the values here and are we all in agreement on the values? It’s just how we’re going to either weigh them or how we’re going to implement them.”*

Leaders reported embracing humility and eschewing blame, encouraging team members to reflect on the factors that lead to a mistake or missed deadline.

*“… if a week goes by and they didn’t do it, I’ll say like, so now what were you thinking when you said that you thought you could get this done (…) it leads to more self-reflection.”*

Holding team members accountable can be especially challenging if these members are not in alignment with team values or if they are unwilling to collaborate. One leader described how an individual’s “attitude” can negatively impact the flow of the team.

*“The attitude’s not there to want to collaborate. So, so I mean what I’ve learned is when there are attitude problems and communication problems, you can try as many team science things as you want. If that attitude hasn’t changed, doesn’t matter. So, the screening process is important…”*

Although it can be challenging to bring together individuals with different expertise and experiences, the leaders interviewed described the importance of establishing shared values, clear expectations, and posing reflective questions to help hold each other accountable. Further, they found that it was important to carefully screen potential team members otherwise they may have to help those team members not in alignment find other opportunities.

In addition to completing tasks and meeting deadlines, leaders also felt it was important to hold team members accountable if they said or did things that negatively impacted other team members. For example, one leader described having a follow-up conversation with a team member when they spoke inappropriately.

*“…this goes back to the leadership component of this for psychological safety when something is said that maybe someone didn’t mean anything by it, but still, there’s a lot there. I don’t let it go, and people look to me to not let it go.”*

Based on their experiences, leaders described being vigilant and watching for these harmful behaviors both on their teams and beyond throughout surrounding external academic environments. They would work to actively “protect” team members from experiencing similar adverse interactions.

## Discussion

In today’s increasingly complex and interconnected world, research is more frequently conducted in interdisciplinary teams bringing together individuals from different backgrounds with varied expertise, perspectives, and problem-solving approaches (Wuchty et al., 2007). The cognitive diversity that arises from such teams has been shown to enhance team performance (Mello and Rentsch, 2015; Wang et al., 2016). However, bias and ineffective team management practices can hinder a team’s ability to leverage the advantages (Carter and Phillips, 2017). Leaders play a vital role in establishing team environments that reinforce psychological safety (Roberson and Perry, 2022). By equipping leaders with skills to effectively foster psychological safety, teams are better positioned to integrate perspectives, unlocking the full potential that diversity offers.

This project was designed to learn from experienced senior leaders as they worked to establish psychological safety in their teams. In addition to identifying specific actions as individual leaders, how they inspired other team members to foster supportive team environments was also of interest. The behaviors described by interviewed leaders fell into five thematic areas: Self-awareness and growth, mentorship and development, amplification and empowerment, communication and transparency, and accountability and integrity. This work serves to complement existing training efforts by offering behavior-level detail. Specific recommendations can then be woven into workshops as teachable skills, enhancing current training frameworks (Bisbey et al., 2021; Brasier et al., 2023a; Sweeney et al., 2023) with actionable insights that deepen leaders’ capacity to translate psychological safety principles into everyday practice.

### Recommendations

Based on what we learned in this study, we provide the following recommendations for promoting psychological safety in teams.

- **Cultivate awareness**—Collaborating effectively with teams requires competencies that support awareness and the exchange of ideas (Bisbey et al., 2021). Thus, strong leadership begins with awareness of self, others, and context. This maps well onto previous research that suggests that leaders need to be aware of how status, hierarchy, and power can silence voices and encourages leaders to model interpersonal risk taking (Edmondson and Bransby, 2023; Jones et al., 2024). Being intentional about how a team is formed further supports awareness by shaping a climate where all voices can be recognized. Leaders who consider team composition, interpersonal fit, and diversity of viewpoints are better positioned to notice power dynamics early and foster environments where individuals feel safe to contribute (Jones et al., 2024).
- **Invest in each team member**—Investing in each team member is foundational to cultivating psychological safety, as individuals feel more willing to contribute when they feel seen, valued, and supported. Leaders who invest time in building authentic relationships can better understand each person’s strengths, challenges, and aspirations, allowing them to tailor support in a way that enhances both individual and team functioning (Brasier et al., 2023a; Edmondson and Bransby, 2023). Developing skills in facilitation can help enhance psychological safety by helping team surface and navigate interpersonal dynamics more effectively (Jones et al., 2024).
- **Make the implicit explicit**—Making the implicit explicit is essential for cultivating psychological safety, as unspoken norms and assumptions can quietly erode trust and hinder open communication. Leaders who encourage transparency and foster open dialogue help surface hidden expectations, challenge inaccurate assumptions, and ensure that decision making processes and roles are clearly understood so no one is caught off guard. Previous research emphasizes that teams thrive when candor is expected and supported, and when leaders create conditions where people feel safe to share concerns, uncertainties, and differing viewpoints (Edmondson and Bransby, 2023; Jones et al., 2024). By making the implicit explicit, leaders not only support psychological safety but also strengthen shared understanding, alignment, and collective effectiveness.
- **Create opportunities for reflexivity**—Reflection is key to growth for both individuals and teams. Leaders can cultivate curiosity and humility by encouraging team members to pause, reflect, and engage in meaningful dialogue about their own assumptions, behaviors, and interactions (Bisbey et al., 2021; Brasier et al., 2023a). This emphasis on reflection aligns with the broader psychological safety literature, which highlights that learning behaviors (e.g., discussing errors, seeking feedback, and reexamining team processes) are central to high performing, adaptive teams (Edmondson and Bransby, 2023). Facilitation practices help teams move through continuous cycles of observation, interpretation, and action that make learning possible. These structured moments of reflection allow teams to surface tensions, recalibrate norms, and adjust their collaborative practices in ways that strengthen psychological safety (Jones et al., 2024). By intentionally creating space for both self-reflection and collective learning, leaders not only invite constructive dialogue but also reinforce a culture where feedback is valued, shared understanding deepens, and team members feel increasingly safe to engage authentically.
- **Share leadership**—Sharing leadership is essential for building well integrated teams, as distributing responsibility empowers individuals to take initiative, contribute expertise, and actively influence team functioning. When leadership is shared rather than concentrated, team members become co-stewards of the norms and expectations that support collaboration, creating a culture of mutual accountability (Brasier et al., 2023a). This aligns with broader psychological safety research, which identifies leadership as a central driver of team climate and emphasizes that teams perform best when members feel empowered to speak up, contribute ideas, and engage in collective problem solving (Edmondson and Bransby, 2023). By sharing leadership, teams distribute the work of noticing, interpreting, and responding to interpersonal dynamics, strengthening everyone’s investment in creating a psychologically safe environment.
- **Work for the greater good**—Working for the greater good requires leaders to think beyond individual achievement and to orient the team toward a shared vision that reflects collective purpose and values (Brasier et al., 2023a). Leaders who model integrity, accountability, and commitment to the broader mission help establish a climate in which team members see their contributions as interconnected and meaningful. This orientation is consistent with research showing that high functioning teams thrive in complex, interdependent environments where people feel safe contributing candidly to a shared goal (Edmondson and Bransby, 2023; Jones et al., 2024). When leaders emphasize the greater good, they help align diverse perspectives, support collaborative engagement, and reinforce the sense of collective responsibility that underpins psychologically safe and mission driven teams.

## Limitations

This study has several limitations that may influence the interpretation and transferability of its findings. While qualitative research prioritizes depth over breadth, the limited number of participants and the singular institutional context may constrain the diversity of perspectives captured. As such, the findings may reflect experiences specific to this setting and may not be readily transferable to other institutions or populations. Data collection occurred during a single time period, offering only a snapshot of participant experiences rather than a longitudinal view. This temporal limitation means that the study may not account for changes over time or evolving contextual factors that could influence the phenomenon under investigation. Additionally, the use of self-reported data introduces the potential for social desirability bias, as participants may have provided responses they perceived as expected, favorable, or socially acceptable rather than fully candid reflections of their experiences. This bias could influence the authenticity of the narratives and shape the themes that emerged. Future research could benefit from engaging a broader and more varied participant pool across multiple institutions and time points to enhance the richness and applicability of the insights.

## Conclusion

This study underscores the transformative power of leadership in cultivating strong team cultures while also complementing and strengthening existing leadership-training efforts. As leadership development programs evolve to prioritize psychological safety as a core competency essential to team success, our work adds nuance by identifying concrete behaviors that can enrich and strengthen these broader institutional initiatives. By embedding the principles of psychological safety into training, evaluation, and organizational culture, institutions can cultivate environments where all voices are heard, valued, and empowered. Ultimately, strengthening these collective training efforts not only benefits individual teams but also contributes to enhancing the culture of research more broadly.

## Data Availability

The datasets presented in this article are not readily available because of privacy and ethical considerations, but the qualitative data that support the findings of this study are available from the corresponding author upon reasonable request. Requests to access the datasets should be directed to wasweeney@wisc.edu.
